# Comparison of characteristics between neuropathic pain and non-neuropathic pain in patients with diabetic carpal tunnel syndrome: A cross-sectional study

**DOI:** 10.3389/fsurg.2022.961616

**Published:** 2022-08-02

**Authors:** Yingnan Liu, Yongqing Zhuang, Ruihong Wei, Zhouyong Tan, Chao Chen, Dazhi Yang

**Affiliations:** ^1^ The Second Clinical Medical College of Jinan University, Shenzhen, Guangdong, China; ^2^ Department of Hand and Microvascular Surgery, Shenzhen People's Hospital, Shenzhen, Guangdong, China; ^3^ Department of Spine Surgery, Shenzhen People's Hospital, Shenzhen, Guangdong, China

**Keywords:** carpal tunnel syndrome, neuropathic pain, diabetic mellitus, cross-sectional study, psychologcal assessment

## Abstract

**Background:**

The aim of the study was to compare the clinical characteristics of diabetic carpal tunnel syndrome between patients with neuropathic pain (NeuP) and non-NeuP.

**Methods:**

We enrolled 276 patients with diabetic carpal tunnel syndrome. Pain symptoms were evaluated using a visual analog scale. Douleur Neuropathique 4, the Neuropathic Pain Symptoms Inventory questionnaire, and the body map were used to assess neuropathic symptoms. Baseline information, clinical manifestations, electrophysiological test results, and psychological status were compared between the neuropathic pain (NeuP) and non-NeuP to identify the risk factor for NeuP occurrence.

**Results:**

Results showed that the degree of pain was more severe in NeuP patients than in nociceptive pain patients (*p* = 0.025). The frequencies of light touch and pinprick were more pronounced in the NeuP group than in the non-NeuP group (light touch: *p *= 0.001; pinprick: *p* = 0.004). There were 48 and 27 NeuP patients with extramedian and proximal spread, respectively, whereas in the non-NeuP group, there were 11 and 9 patients, respectively (*p* = 0.03). Electrophysiological results showed that patients in the NeuP group exhibited greater sensory nerve conduction velocity impairment compared with the non-NeuP group (*p* = 0.033). Pain Catastrophizing Scale total scores of the NeuP group were significantly higher than those of the non-NeuP group (*p* = 0.006).

**Conclusion:**

Of the 276 diabetic carpal tunnel syndrome patients studied, the majority had NeuP. Furthermore, light touch, electrophysiological test results, and psychological factors were found to be related to NeuP occurrence in patients with diabetic carpal tunnel syndrome.

## Introduction

Carpal tunnel syndrome is the most common peripheral nerve entrapment disease with an incidence as high as 10% in the general population (1). The primary symptoms of carpal tunnel syndrome include paresthesia and neuropathic pain (NeuP) in the median territory, weakness of hand grasp, and thenar wasting. NeuP, especially nocturnal pain, is the primary complaint of patients with carpal tunnel syndrome. Pain symptoms directly and negatively impact the sleep quality and hand function of patients, which results in psychological states of anxiety and depression (2).

The International Association for the Study of Pain (IASP) defines NeuP as “pain caused by a lesion or disease of the somatosensory nervous system.” The prevalence of NeuP in carpal tunnel syndrome patients varies from 31% to 80% across studies (3–5). Researchers have dedicated significant effort to determining the characteristics of NeuP in patients with carpal tunnel syndrome. Matesanz et al. reported that the severity of NeuP is associated with more pronounced deficits in emotional well-being and sleep quality (3). Oteo-Alvaro and Marin revealed that numbness/tingling, pain intensity, and neurologic affectation are risk factors for NeuP (4). Moreover, Sonohata et al. found that carpal tunnel release can alleviate NeuP (5).

Diabetes mellitus is a shared risk factor for NeuP and carpal tunnel syndrome (6), and NeuP is often the most pronounced manifestation of carpal tunnel syndrome. Carpal tunnel syndrome and diabetes mellitus have synergistic effects on median nerve injury (7–9). Thus, carpal tunnel syndrome, NeuP, and diabetes mellitus may interact to form a mutual response to the progress of the diseases. Therefore, patients with NeuP who have diabetic carpal tunnel syndrome are likely to present with characteristics that are distinct from those with nondiabetic carpal tunnel syndrome. Understanding the specific symptoms of this subgroup of carpal tunnel syndrome patients could enable more accurate diagnosis and the development of more focused therapies. However, analyses of the characteristics of NeuP in patients with diabetic carpal tunnel syndrome are scarce.

In our clinical practice, we have observed differences between NeuP patients and non-NeuP patients of carpal tunnel syndrome in terms of demographic information, clinical manifestation, and psychological state. Therefore, in the current study, we enrolled 276 diabetic carpal tunnel syndrome patients and divided them into two groups according to the pain symptoms .Then, we compared the clinical characteristics between these two groups to identify possible risk factors for the occurrence of NeuP in patients with diabetic carpal tunnel syndrome. The characteristics of NeuP in diabetic carpal tunnel syndrome patients would provide the hints for the hand surgeons to take some interventions earlier.

## Patients and methods

This study was approved by the Ethical Committee of Shenzhen People's Hospital.

### Study population

All participants diagnosed with both carpal tunnel syndrome and diabetes mellitus in the department of hand and microvascular surgery which focused on the peripheral nerve surgery in our hospital, between June 1, 2020 and June 2, 2021, who provided written informed consent and were willing to participate, were recruited into this cross-sectional study. We recruited 276 unilateral carpal tunnel syndrome patients (216 women and 60 men) who were screened according to the inclusion and exclusion criteria. Eligible participants included patients who were aged over 18 years, and who were diagnosed with both carpal tunnel syndrome and diabetes mellitus (Type 1 or Type 2) based on symptoms, physical examinations, and electrophysical tests. The exclusion criteria were as follows: bilateral carpal tunnel syndrome, acute complications of diabetes mellitus (e.g., renal failure, foot ulcers, and severe infection), other NeuP diseases (e.g., peripheral nerve lesions, brain and spinal cord lesions, thyroid dysfunction, and multiple sclerosis), and inability to read or write Chinese (illiterate or an ethnic minority). Patients diagnosed with bilateral carpal tunnel syndrome were excluded because they always presented with different clinical manifestations between the right and left hands, creating difficulties in the data analysis.

### Definite diagnosis of neuropathic pain and carpal tunnel syndrome

Firstly, the visual analog scale (VAS) was used to evaluate whether patients with diabetic carpal tunnel syndrome presented with pain symptoms. Patients after the VAS scale evaluation were diagnosed using the Douleur Neuropathique 4 (DN-4) scale, which is the common diagnosis standard for NeuP (10). The DN-4 scale was used for the definite diagnosis of NeuP in patients with diabetic carpal tunnel syndrome. The questionnaire consists of ten questions evaluating sensory descriptors, and a sensory examination assessing tactile sensation, pinprick, and allodynia. Patients with a DN-4 score of ≥4 were diagnosed with NeuP (11).

Diagnosis of carpal tunnel syndrome was based on clinical manifestations, physical examinations, and electrophysiological tests. The symptoms of carpal tunnel syndrome were numbness or tingling in the median nerve distribution for at least one month. The physical examination for carpal tunnel syndrome detected paresthesia with or without thenar atrophy. The electrophysiology test for carpal tunnel syndrome involved detection of delayed median nerve terminal latency (>3.6 ms). Patients with symptoms but the normal electrophysiology were also diagnosed with carpal tunnel syndrome (12). Patients with extramedian and proximal spread symptoms were also evaluated using spinal MRI or computed tomography to exclude cervical spine-related diseases.

### Baseline characteristics

We reviewed the medical records from the hospital database of all participants to collect basic information, including sex, age, height, weight, educational status, and living habits. We also reviewed the duration of clinical manifestations, including median symptom duration and diabetes symptom duration. Levels of glycosylated hemoglobin (HbA1c) were recorded to determine the severity of diabetes during the past 3 months.

### Clinical manifestations

To evaluate the clinical symptoms of diabetic carpal tunnel syndrome, we used the Boston Carpal Tunnel Syndrome Questionnaire (BCTQ). The BCTQ comprises a symptom severity score (BCTQ-S) and a functional status score (BCTQ-F), which represent symptom severity and functional deficit, respectively, after median nerve compression (13). The BCTQ-S consists of 11 questions related to symptom severity, whereas the BCTQ-F consists of eight questions on hand function during daily activities. Each question is rated on a five-point scale from 1 (none) to 5 (most severe), and the average score of each item is calculated. The validated translated Chinese version of the BCTQ was used for the current study (12).

In addition to the BCTQ, each participant underwent a physical examination of the sensory and motor function of the median nerve. Light touch and pinprick were evaluated using cotton wool and a neurotip on the palm side of the index finger. The sensation was recorded as normal or reduced compared with the same finger on the other hand. The Tinel sign test involves tapping over the median nerve at the entrance of the carpal tunnel, and is considered positive if the patient senses numbness, tingling, and shooting pains in the thumb, index finger, middle finger, the radial half of the ring finger, and the palm. The Phalen test involves flexion of the wrist to the unforced extreme angle for 60 s, and a positive test is recorded if numbness, tingling, and shooting pains are experienced or exaggerated at the distribution of the median nerve. We also evaluated the muscle strength of the abductor pollicis brevis muscle according to the Medical Research Council scale.

### Electrophysiological test

The electrophysiological test was performed using the KEY POINT (Alpine Biomed, Denmark) system. The hand temperature was maintained above 31°C. Median nerve motor function was evaluated by median nerve terminal latency and the compound muscle action potential (CMAP), which were recorded from the abductor pollicis brevis muscle while applying stimulation from the wrist to antecubital fossa. Median nerve sensory function was evaluated by sensory nerve conduction velocity (SNCV), which was recorded from the wrist while stimulating the index finger.

### Pain evaluation

The VAS was used to evaluate the degree of general pain of participants. Patients selected a number that corresponded to their recently experienced pain, on a scale from 0 (no pain) to 10 (the worst pain). We then applied the Neuropathic Pain Symptom Inventory (NPSI), which is a widely used tool for characterizing NeuP symptoms (14, 15). NPSI is a self-reported questionnaire that is specifically designed to evaluate NeuP symptoms and has been validated in more than 50 different languages, including Chinese. This questionnaire comprises five subgroups that represent four aspects of NeuP: burning spontaneous pain, pressing spontaneous pain, paroxysmal pain, evoked pain, and paresthesia/dysesthesia. We also used a combination of the VAS and the body map to indicate pain distributions involved in diabetic carpal tunnel syndrome. We determined and analyzed the involved nerve distribution areas, which included fingers, palm, extramedian distributions, and proximal areas. After comparing the pain distributions between the NeuP group and the non-NeuP group, we investigated the characterstics of neuropathic pain distribution.

### Psychological status evaluation

The pain catastrophizing scale (PCS) consists of 13 self-reported items that measure pain-related catastrophizing phenomena in the context of actual or anticipated pain (16). The PCS questionnaire was used to evaluate patients' psychological status. The PCS measures catastrophizing phenomena along three dimensions: rumination, magnification, and helplessness (17). It has been widely applied to evaluate various pain conditions, such as low back pain, diabetic pain, NeuP, and stroke pain, and has been shown to be related to pain outcomes. We compared PCS scores between NeuP and non-NeuP groups to determine the pain catastrophizing effect of NeuP in patients with diabetic carpal tunnel syndrome.

### Statistical analysis

Student's t-tests were used to compare the continuous variables between groups, chi-square tests were used to analyze the rate and constituent ratio index, the Shapiro-Wilk test was used to test data normality, and non-normal data were analyzed using the Wilcoxon test. We calculated means and standard deviations for continuous data and frequencies for categorical data. The SPSS software (version 23.0, IBM, New York) was used for all data analyses. We used a *p* < 0.05 to signify significance.

## Results

### Demographic factors between the two groups

The demographic information is listed in Tables 1. A total of 198 (71.7%) participants with a DN-4 score of ≥4 were classified into the NeuP group, and the remaining 78 (28.3%) participants with a DN-4 score of <4 were classified into the non-NeuP group. Among the non-NeuP group, half (*n* = 39) of the patients were classified as having no pain, and the other half were classified as having nociceptive pain. We also tested the VAS scores of the NeuP and non-NeuP groups. The VAS score of the NeuP group was 5.5 ± 3.2, which was significantly higher than that of the non-NeuP group (*p* = 0.021). We also compared VAS scores between the NeuP and nociceptive pain patients, and found that the degree of pain of NeuP patients was greater than that of nociceptive pain patients (*p* = 0.025). Notably, we also found that the median symptom duration of the NeuP group was 4.12 ± 2.11 months, whereas the median symptom duration of the non- NeuP group was 8.4 ± 3.22 months (*p *= 0.014). This indicated that diabetic carpal tunnel syndrome patients with NeuP experienced pain symptoms over a shorter period. Finally, we compared the neuropathic pain proportion between the Type 1 or Type 2 diabetic mellitus and the results showed no significant difference (*p *= 0.423).

**Table 1:** Demographic information of the two groups.

	All	No NeuP	NeuP	*P-*value
No of participants, *n* (%)	276	78 (28.3%)	198 (71.7%)	
VAS	4.5 ± 3.6	1.8 ± 1.2	5.5 ± 3.2	**0.021**
Gender, *n* (%)				0.760^a^
Female	216(78.26%)	59(75.6%)	157(79.3%)	
Male	60(21.84)	19(24.4%)	41(20.7%)	
Age, years	58.0 ± 19.2	52.2 ± 10.2	60.3 ± 14.9	0.133
Mean height, cm	163.6 ± 21.3	159.3 ± 12.2	165.4 ± 21.0	0.159^b^
Mean weight, kg	74.3 ± 26.3	69.3 ± 21.5	76.4 ± 23.9	0.062
Educational degree, years	9.0 ± 3.4	8.1 ± 2.2	9.3 ± 3.11	0.681
Living habits				
Smoking, *n* (%)	190(68.8%)	51(65.4%)	139(70.0%)	0.512^a^
Cigarettes per day	8.2 ± 7.9	7.4 ± 6.3	8.5 ± 7.6	0.534^b^
Drinking, *n* (%)	131(47.5%)	30(38.5%)	101(51.0%)	0.214^a^
Alcohol per day, gram	35.3 ± 32.6	23.5 ± 11.4	40.0 ± 34.1	0.256
Median symptom duration, mo	5.3 ± 2.8	8.4 ± 3.2	4.1 ± 2.1	**0.014**
Diabetes symptom duration, mo	32.3 ± 13.5	30.1 ± 7.1	33.1 ± 12.3	0.331^b^
HbA1c, %	7.0 ± 1.1	6.9 ± 1.1	7.1 ± 0.9	0.546
Types of DM, *n* (%)				
Type 1 DM	11(3.99%)	4(36.36%)	7(63.64%)	0.423^a^
Type 2 DM	265(96.01%)	74(27.92%)	191(72.08%)	

Figures of vas score, age, educational degree, mean height, mean weight, cigarettes/alcohol per day, symptom duration and HbA1c were presented with Mean ± SD. Other figures were presented with numbers (percentage).

VAS: Visual Analogue Scale, HbA1c: glycosylated hemoglobin, DM: diabetic mellitus.

Bold values meant *P*-value <0.05 and the figures are statistically different.

^a^ Chi-square test.

^b^ Mann-Whitney test.

### Paresthesia is related to the occurrence of neuropathic pain

Comparisons of clinical manifestations between the two groups are shown in Table 2. We used the BCTQ to assess symptom severity and limb function. Results showed that there was no significant difference between the two groups in terms of functional status (*p* = 0.391). However, symptom severity was greater in the NeuP group than in the non-NeuP group (*p* = 0.037).

**Table 2:** Clinical presentations of the two groups.

	No NeuP	NeuP	*P*-value
Boston Carpal Tunnel Questionnaire			
Symptom Severe Score	2.1 ± 0.7	2.9 ± 1.1	0.037
Functional Status Score	2.4 ± 0.6	2.8 ± 0.5	0.391
Clinical examination *n* abnormal (%)			
Light tough	10(12.8%)	89(44.9%)	**0** **.** **001** ^a^
Pinprick	15 (19.2%)	103(52.0%)	0.004
Phalen test	53 (67.9%)	135 (68.2%)	0.771
Tinel sign	32 (41.0%)	98(49.5%)	0.514
Compression sign	29(37.2%)	85(43.0%)	0.122
Muscle strength			
MRC3	0(0.00%)	7(3.5%)	0.486^a^
MRC4	15(19.2%)	14(7.1%)	0.542
MRC5	63(80.8%)	177(89.4%)	0.061
Thenar atrophy	15 (19.2%)	35 (19.7%)	0.451^a^

The data sets of symptom severe score and functional status score were expressed as Mean ± SD.

The data sets of other clinical symptoms were expressed as *n* (%).

Bold values meant *P*-value <0.05 and the figures are statistically different.

MRC, Medical Research Council Muscle Strength Scale.

^a^ Chi-square test.

Furthermore, the frequencies of light touch and pinprick were more pronounced in the NeuP group than in the non-NeuP group (light touch: *p *= 0.001; pinprick: *p* = 0.004). There were no significant differences in Phalen and Tinel signs, which are considered two important physical examination components in the diagnosis of carpal tunnel syndrome, between the two groups.

### Electrophysiological tests provide clues for neuropathic pain

Electrophysiological results showed that patients in the NeuP group exhibited more SNCV impairment than the non-NeuP group (*p* = 0.033), which provides evidence that nerve injury is related to the occurrence of NeuP (Table 3). Although the CMAP did not significantly differ between the two groups, the median nerve terminal latency of the NeuP group was more prolonged than that of the non-NeuP group (CMAP: *p* = 0.341; median nerve terminal latency: *p* = 0.043; Table 3).

**Table 3:** Electrophysiologic tests of the two groups.

	No NeuP	NeuP	*P*-value
Sensory nerve conduction velocity(m/s)	34.7 ± 8.9	30.5 ± 7.9	0.033^a^
Median nerve terminal latency (ms)	5.1 ± 1.5	6.1 ± 1.9	0.043
CMAP (mv)	5.6 ± 3.4	5.8 ± 3.2	0.341

Data sets of the electrophysiologic tests were presented with Mean ± SD.

CMAP, compound muscle action potential.

^a^ Mann-Whitney test.

### Neuropathic pain patients tended to experience more extramedian and proximal spread symptoms

After determining the possible risk factors for NeuP in diabetic carpal tunnel syndrome patients, we analyzed the symptom characteristics of NeuP in both groups (Tables 4, 5).

**Table 4:** Symptomatic comparison of neuropathic pain symptoms between two groups.

Type	Subgroup	No NeuP		NeuP		
		N	Score	N	Score	P
Burning (Superficial)	Spontaneous Pain	4 (5.1%)	1.8 ± 0.4	56 (28.3%)	4.8 ± 2.1	0.041^a^
Pressing (Deep)	Spontaneous Pain	4 (5.1%)	1.8 ± 0.4	20 (10.1%)	4.1 ± 2.3	0.004
	Paroxysmal Pain	3 (3.8%)	2.4 ± 1.0	64 (32.3%)	5.3 ± 2.9	0.241
	Evoked Pain	3 (3.8%)	3.1 ± 2.0	43 (21.7%)	3.3 ± 2.6	0.835
	Paresthesia/Dysesthesia	56 (71.8%)	3.1 ± 2.5	165 (83.33%)	6.7 ± 2.0	0.001

Figures of pain scores of different subgroups were presented with Mean ± S.

^a^ Mann-Whitney test.

**Table 5:** Comparison of symptoms distributions between different groups.

Fingers affected, *n* (%)	No NeuP	NeuP	*P*-value
1	6 (7.7%)	10(5.1%)	0.03^a^
2	35 (44.9%)	40(20.2%)	
3	8(10.3%)	14(7.1%)	
4	9(11.5)	59(29.8%)	
Extra median spread	11(14.1%)	48(24.2%)	
Proximal spread	9(11.5%)	27(13.6%)	

^a^ Chi-square test.

Firstly, we found that the symptom distribution of both groups differed significantly. There were 48 and 27 patients in the NeuP group who had extramedian and proximal spread, respectively, whereas 11 and 9 patients in the non-NeuP group had extramedian and proximal spread, respectively (*p* = 0.03). This suggests that NeuP was not strictly confined to the median nerve distribution. In the non-NeuP group, the majority of patients (44.9%) had symptoms involving two fingers. However, in the NeuP group, most patients (37.8%) had symptoms around the area outside of the median nerve. The analysis of the pain symptoms of diabetic carpal tunnel syndrome patients using the NPSI showed that the most predominant symptoms were paresthesia/dysesthesia, which was observed in 83.33% of patients in the NeuP group and 71.80% of patients in the non-NeuP group. However, we also found that the severity of paresthesia/dysesthesia in the NeuP group was higher than in the non-NeuP group (*p *= 0.001). In addition, the NeuP group had more deep and superficial spontaneous pain than the non-NeuP group (deep spontaneous pain: *p* = 0.004; superficial spontaneous pain: *p* = 0.041).

### Pain catastrophization always accompanies neuropathic pain patients

Given that NeuP affects the psychological status of carpal tunnel syndrome patients, we also evaluated PCS scores between the two groups (Table 6). The PCS total score of the NeuP group was significantly higher than that of the non-NeuP group (*p* = 0.006). The rumination score of the NeuP group was 4.7 ± 3.4, whereas that of the non-NeuP group was 2.6 ± 1.2 (*p* = 0.03). The magnification score of the NeuP group was also significantly higher than that of the non-NeuP group (*p* = 0.04). The helplessness score of the NeuP group was markedly higher than the non-NeuP group, although the difference was not significant (*p* = 0.062).

**Table 6:** Comparison of psychologic factors in two groups in diabetic CTS patients.

Emotional well being	No NeuP	NeuP	*P*-value
PCS total	8 ± 2.3	14 ± 4.2	0.006^a^
Rumination	2.6 ± 1.2	4.7 ± 3.4	0.03
Magnification	3.0 ± 1.0	5.5 ± 2.3	0.04
Helplessness	2.8 ± 1.5	4.0 ± 2.3	0.062

PCS, Pain Catastrophizing Scale.

^a^ Mann-Whitney test.

## Discussion

Diabetes mellitus is considered a putative risk factor for carpal tunnel syndrome and is also a shared risk factor for NeuP (18, 19). There has been extensive research investigating the characteristics of NeuP in patients with carpal tunnel syndrome and diabetic carpal tunnel syndrome (3, 4, 20). However, few studies have elucidated the risk factors, symptom characteristics, and related psychological factors of patients with both diabetic carpal tunnel syndrome and NeuP. Therefore, we studied 276 diabetic carpal tunnel syndrome patients and divided them into a NeuP and non-NeuP groups according to DN-4 scale scores. We compared clinical data between the two groups to obtain a better understanding of this subgroup of patients with carpal tunnel syndrome.

The prevalence of NeuP in this cohort of patients was 71.7%, which indicated that the majority of the diabetic carpal tunnel syndrome patients experienced NeuP. Previous studies have also reported similar results. Oteo-Alvaro and Marin reported that 76.7% of patients with carpal tunnel syndrome have NeuP (4). Matesanz et al. also reported a prevalence as high as 80% (3). Moreover, Esma reported that 72.7% of carpal tunnel syndrome patients develop NeuP symptoms. Taken together, we found that the occurrence rate of NeuP in diabetic carpal tunnel syndrome patients is comparable to that in all carpal tunnel syndrome patients.

We then compared demographic data between the two groups, which included sex, age, height, weight, educational status, living habits, symptom duration, and HbA1c level. Of these risk factors, median symptom duration showed a significant difference between the non-NeuP and NeuP groups. The median symptom duration of the NeuP group was shorter than that of the non-NeuP group, which indicated that the NeuP patients developed NeuP at an earlier period. This contradicts the common notion that severity of pain symptoms is correlated with disease duration. A possible explanation is that the median nerve injury of the NeuP group was more severe than that of the non-NeuP group; therefore, patients in the NeuP group developed pain symptoms earlier (21). Although differences in alcohol use and the amount of alcohol consumed did not reach significance, the difference between the NeuP and non-NeuP groups is nevertheless notable because alcoholic neuropathy may contribute to NeuP.

Light touch is a prominent symptom of diabetic carpal tunnel syndrome (1). We found that the incidence of abnormal light touch in the NeuP group was significantly higher than that in the non-NeuP group. Previous studies have shown that sensory function is related to the pain phenomenon (22, 23). Different sensory functional deficits may have different underlying mechanisms. In addition, we found that the SNCV of the NeuP group was significantly slower than that of the non-NeuP group. However, the CMAP did not differ significantly between the two groups. The electrophysiological results showed that the sensory nerve is predisposed to be affected in the NeuP group, whereas motor nerve function does not play a central role in the occurrence of NeuP. The electrophysiological results also suggested that the slower SNCV of the NeuP group accounts for the prolonged median nerve terminal latency.

Comparison of the degree of pain showed that spontaneous pain, both superficial and deep, was more severe in the NeuP group compared with the non-NeuP group. Paresthesia and dysesthesia were also significantly different between the two groups. These results were consistent with the clinical manifestations and electrophysiological results.

In addition to pain symptoms, we investigated pain distributions in the two groups. Results showed that the NeuP group had more extramedian and proximal distributions than the non-NeuP group. It has been reported that central mechanisms are also involved in NeuP in patients with carpal tunnel syndrome (24–26). Our results confirmed that central mechanisms play a vital role in diabetic NeuP. The central mechanisms of carpal tunnel syndrome include sensitization and descending facilitation. Previous studies have reported that hyperalgesia, allodynia, and wind-up in extramedian territories are the main sensitization presentations (27–29). In our study, we found that sensitization also correlated with the occurrence of NeuP in diabetic carpal tunnel syndrome patients.

Previous studies have reported that carpal tunnel syndrome patients' mindset and pain catastrophization are related to outcomes (2). We revealed that the NeuP symptoms of diabetic carpal tunnel syndrome patients involved pain catastrophization (30). The underlying mechanism may be that nocturnal pain exerts a negative effect on sleep quality. Moreover, declining grip strength may hinder the work and daily life abilities of patients (31). Insomnia and other life disturbances may also predispose patients to catastrophize, which subsequently creates a vicious cycle (32). Furthermore, pain catastrophization is considered to be related to central mechanisms. The pain catastrophization process is involved in mediating the association between central sensitization and pain expectancy (33). Pain catastrophization also exerts harmful and maladaptive effects on the social environment, and amplifies the central processing of pain (34). Therefore, sociopsychological interventions should be developed to disrupt the pain catastrophization process.

Several limitations of the current research warrant discussion. This was a croos-sectional study; thus,we could only provide potentially related factors for the occurrence of NeuP in diabetic carpal tunnel syndrome patients. Determining the exact cause-effect relationship requires further case-control and cohort studies. In addition, we excluded bilateral carpal tunnel syndrome patients because bilateral clinical manifestations present difficulties in making comparisons. However, bilateral symptoms may also be a factor relevant to NeuP. Therefore, in future research, we encourage case-control and cohort studies that include bilateral carpal tunnel syndrome patients.

## Conclusion

Our cross-sectional study of 276 diabetic carpal tunnel syndrome patients revealed that NeuP accounted for the majority of those patients. A total of 198 (71.7%) participants were diagnosed as NeuP. Light touch and electrophysiological test results were related to the occurrence of NeuP. Patients in the NeuP group tended to experience more extramedian and proximal symptoms. Moreover, pain catastrophization was associated with the occurrence of NeuP in patients with diabetic carpal tunnel syndrome.

## Data Availability

The raw data supporting the conclusions of this article will be made available by the authors, without undue reservation.
